# Decoding tumor angiogenesis: pathways, mechanisms, and future directions in anti-cancer strategies

**DOI:** 10.1186/s40364-025-00779-x

**Published:** 2025-04-18

**Authors:** Xueru Liu, Juan Zhang, Ting Yi, Hui Li, Xing Tang, Dan Liu, Daichao Wu, Yukun Li

**Affiliations:** 1 Department of Assisted Reproductive Centre, Zhuzhou Hospital Affiliated to Xiangya School of Medicine, Central South University, Zhuzhou, 412000 Hunan China; 2 Department of Trauma Center, Zhuzhou Hospital Affiliated to Xiangya School of Medicine, Central South University, Zhuzhou, 412000 Hunan China; 3 Laboratory of Structural Immunology, Department of Hepatopancreatobiliary Surgery, Hengyang Medical School, The First Affiliated Hospital, University of South China, Hengyang, 421001 Hunan China

**Keywords:** Tumor Angiogenesis, Cancer, Tumor microenvironment, Endothelial, Vascular targeting, Molecular Pathways, Anti-angiogenic therapy

## Abstract

Angiogenesis, a crucial process in tumor growth and metastasis, necessitates targeted therapeutic intervention. This review reviews the latest knowledge of anti-angiogenesis targets in tumors, with emphasis on the molecular mechanisms and signaling pathways that regulate this process. We emphasize the tumor microenvironment's role in angiogenesis, examine endothelial cell metabolic changes, and evaluated potential therapeutic strategies targeting the tumor vascular system. At the same time, we analyzed the signaling pathway and molecular mechanism of tumor angiogenesis in detail. In addition, this paper also looks at the development trend of tumor anti-angiogenesis drugs, including their future development direction and challenges, aiming to provide prospective insight into the development of this field. Despite their potential, anti-angiogenic therapies encounter challenges like drug resistance and side effects, necessitating ongoing research to enhance cancer treatment strategies and the efficacy of these therapies.

## Introduction

Tumor is a new growth formed by the proliferation of local tissue cells in the body due to various tumorigenic factors. It has the characteristics of rapid reproduction, strong metabolism and tenacious vitality, and its requirement for oxygen and nutrients is higher than that of normal tissue cells [1]. Angiogenesis is the process of developing a new vascular network through the original vascular system. The core mechanism includes the degradation of the vascular basement membrane, the activation and proliferation of endothelial cells (ECs), and cell migration, and finally the reconstruction of the vascular network [2, 3]. This process is important for tissue repair and embryonic development in physiology and is closely related to tumor growth and inflammatory response in pathology, involving diverse cells and molecules [4]. ECs in the inner blood vessel wall are crucial for angiogenesis and vascular homeostasis, facilitating oxygen and nutrient supply to meet the body's metabolic demands. During angiogenesis, endothelial progenitor cells (EPCs) are recruited, differentiate into mature vascular ECs in response to local stimuli, and form blood vessels [5]. Neovascularization is crucial for tumor growth and metastasis, facilitating nutrient transport and metabolic waste removal from tumor cells. Immature neovascularization induces hypoxia and acidosis in the tumor microenvironment, accelerating tumor growth and metastasis [6, 7]. It’s a self-reinforcing cycle [8, 9]. Angiogenesis is crucial for tumor growth, metastasis, and prognosis.

Anti-angiogenesis therapy is a novel approach for treating tumors. Angiogenesis is now a crucial target in cancer therapy, focusing primarily on the VEGF/VEGFR, PDGF/PDGFR, EGF/EGFR, and FGF/FGFR signaling pathways [10]. In recent years, anti-tumor angiogenesis research has shifted from single-target anti-angiogenic drugs to combination therapies, immunotherapies, and multi-target anti-angiogenic agents due to adverse effects, increasing drug resistance, off-target toxicity, and tumor recurrence [10–14]. In addition, many metabolites in natural drugs have been found to possess high anti-cancer activity and low side effects, which deserve further detect [15].

While anti-angiogenic therapy offers significant survival benefits by reducing tumor size and extending survival time, it also presents challenges, acting as a double-edged sword. However, adverse effects like headache, hypertension, proteinuria, bleeding, thrombosis, and thrombocytopenia have brought great challenges to its clinical application [16]. Maximizing therapeutic effects while minimizing side effects is a significant challenge in clinical anti-angiogenic therapy. Resistance to anti-angiogenic drugs is common in the treatment of cancer patients, which significantly affects the prognosis of patients. At the same time, the therapeutic effect of single-agent pro-angiogenic therapy is relatively limited, which poses a major challenge in the current field. Anti-angiogenic drugs such as Bevacizumab face resistance challenges in clinical application. The main mechanisms include: redundancy of VEGF signaling (such as up-regulation of VEGFC and PIGF); Changes in biological characteristics of tumor cells (invasion and metastasis, enhanced stem cell characteristics, autophagy and metabolic reprogramming); The pro-angiogenic effects of bone marrow-derived cells (MDSCs, TAMs) and local stromal cells (pericytes, CAFs); And alternative models of angiogenesis (co-selection and mimicry) [17]. Consequently, researchers have concentrated more on enhancing therapeutic efficacy and reducing drug resistance [17, 18]. At present, a variety of multi-targeted angiogenesis inhibitors, such as Sorafenib [19], Lenvatinib and Vorolanib [20, 21], have been developed for the treatment of tumors. The combination of anti-angiogenesis drugs and immunotherapy exhibits a synergistic effect. In addition, numerous pivotal clinical trials have evaluated the combination of angiogenesis inhibitors with traditional cancer therapies such as chemotherapy, radiotherapy, biotherapy, immunotherapy, adoptive cell therapy, and cancer vaccines in patients with various cancer types [22]. Ongoing research into the molecular mechanisms of angiogenesis and the development of novel therapeutic agents targeting these pathways offers hope for more effective cancer treatments with fewer side effects.

This review outlines the process and characteristics of tumor angiogenesis, its impact on tumor growth, spread, invasion, and metastasis, the composition of the tumor microenvironment and its influence on angiogenesis, the metabolic changes of ECs within the tumor microenvironment, and the potential for targeting tumor blood vessels. This review also examines the signaling pathways and molecular mechanisms driving tumor angiogenesis. In addition, we also outline some anti-angiogenic drugs and their mechanisms of action.

## The pathophysiological process of tumor angiogenesis

In 1971, Professor Folkman suggested that angiogenesis is crucial for tumor growth and metastasis, offering a new theoretical foundation for anti-tumor angiogenesis drugs [23]. Tumor angiogenesis is the process where new blood vessels form from existing ones, supplying nutrients, oxygen, and a cellular network essential for tumor growth [23–27]. The complex vascular network facilitates tumor cells entering the bloodstream and metastasizing to distant body sites. Tumor angiogenesis is a complex process regulated by pro-angiogenic and anti-angiogenic factors within solid tumors [28, 29]. When pro-angiogenic factors outnumber anti-angiogenic factors, tumors will form new blood vessels. Tumor growth and spread are largely dependent on tumor blood vessels. Once a tumor is formed, with its continuous growth, when its diameter exceeds 1–2 mm, if it cannot timely neovascularization to provide oxygen and nutrition, it is difficult to continue to grow [30, 31]. Benign tumor cells exist in a quiescent state and are affected when it is difficult to obtain an adequate blood supply. However, when angiogenesis of dormant tumor cells is activated and secreted factors induce ECs sprouting and chemotaxis to form tumor masses, an"angiogenic switch"occurs [32]. The angiogenic switch is primarily activated by the secretion of growth factors and cytokines, oncogene activation, tumor suppressor gene inactivation, and tumor-associated hypoxia [33].

**Fig. 1:**
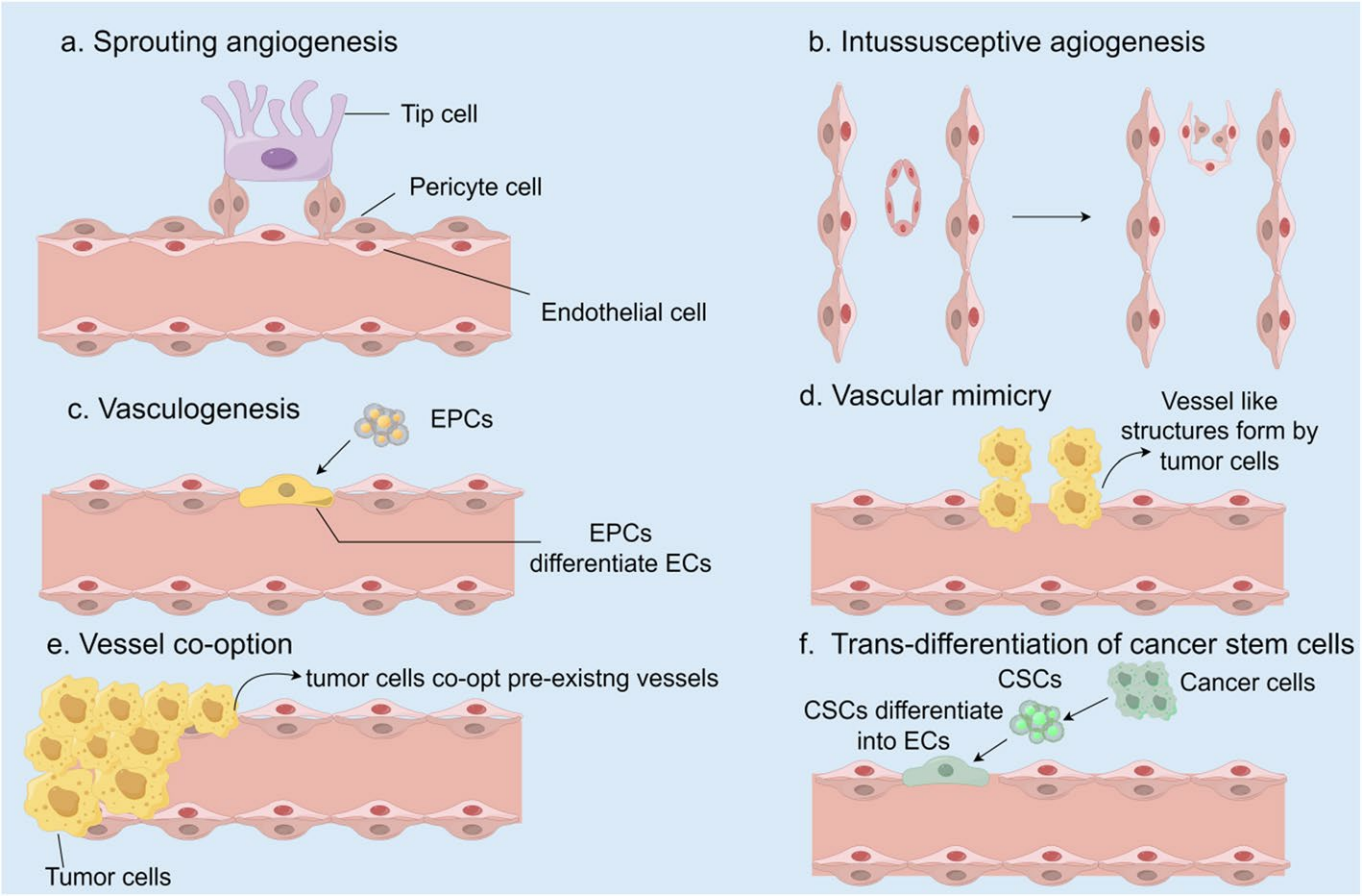
The most prevalent pattern of tumor angiogenesis. **a** Sprouting angiogenesis: this process involves the growth of new blood vessels from the existing vasculature; **b** Intussusceptive angiogenesis: the lumen of the existing blood vessel splits and eventually the blood vessel splits into two; **c** Vasculogenesis: It refers to the process of angiogenesis from scratch, the differentiation of EPCs in the bone marrow into ECs, and finally the construction of a new vascular system; **d** Vascular mimicry: tumor cells form a vascular structure that directs oxygen and nutrients to the tumor tissue; **e** Vessel co-option: tumor cells utilize the existing vasculature for their own growth needs instead of inducing new blood vessels; **f** Trans-differentiation of cancer stem cells (CSCs): CSCs transform into ECs, which in turn participate in the formation of new vascular network supporting tumor growth. In normal tissues and tumors, the first three patterns were common. The subsequent three were specifically associated with tumor angiogenesis

Tumor angiogenesis can arise through various mechanisms, each distinct in its process. Key processes include sprouting angiogenesis, intussusceptive angiogenesis, vasculogenesis, vasculogenic mimicry, vessel co-option, and trans-differentiation of cancer stem cells (Fig. 1) [34]. The formation of sprouting vessels is regarded as the most representative step in the physiological and pathological process of angiogenesis.

Sprouting angiogenesis. It is the earliest discovered way of tumor angiogenesis. This process, which includes steps such as endothelial cell activation, matrix degradation, cell invasion, proliferation, migration, formation of vascular lumen, and stabilization of new blood vessels, is tightly controlled by positive and negative regulators [35]. Among them, Vascular endothelial growth factor (VEGF) plays a crucial role in forming new blood vessel branches [34, 36], involving tip cell migration and stem cell proliferation to form lumen-like structures that connect with other vessels.

Intussusceptive angiogenesis. It is a process first observed in pulmonary capillary remodeling [37, 38], in which blood vessels split longitudinally into two capillaries. Intussusceptive angiogenesis is a rapid and highly efficient process that even takes only a few minutes. The molecular mechanisms of intussusceptive angiogenesis is unknown, but VEGF seems to induce this process [39], indicating that VEGF signaling inhibitors might effectively block it.

Vasculogenesis. Tumor vasculogenesis involves not only the recruitment of bone marrow-derived cells, especially EPCs, which can transform into ECs to construct the neovascularization network required to support the continuous growth of tumors. This complex process is critical for tumor development and spread, and therefore can also be a target in anti-tumor therapeutic strategies [34]. EPCs have excellent reproductive ability, self-renewal, and participate in neovascularization and promote endothelial tissue repair [40, 41].

Vascular mimicry (VM). During VM, the malignant tumor cells themselves undergo morphological changes, and the tumor cells extend to form lumen-like structures resembling blood vessels, which then connect with preexisting blood vessels. This angiogenesis mode is completely different from the traditional tumor angiogenesis mode. The tumor is not attached to vascular ECs, which can realize direct contact with blood and effectively promote the delivery of red blood cells and oxygen to the tumor area [42]. Under hypoxic conditions, epithelial-mesenchymal transition is accelerated, resulting in the generation of a network of channels formed by cancer cells in an extracellular matrix (ECM) -rich environment. This mechanism not only ensures the stability of blood supply to the tumor, but also promotes its effective communication with the peripheral vascular system [42]. This is a rare biological phenomenon that has only been observed in a limited number of tumor types, but it has shown a significant correlation with tumor drug resistance. Therefore, it is of great theoretical and application value to deeply study and explore the molecular mechanism of VM, as well as to identify and target specific molecular pathways that interfere with this process [43–45].

Vessel co-option. Vessel co-option is similar to VM, which represents a non-angiogenic process. Tumor cells not only rely on the pre-existing vascular network for growth and spread [46], but also migrate along the blood vessels to penetrate the surrounding tissues to obtain the oxygen and nutrients they need [47]. Vessel co-option has become a crucial resistance mechanism in anti-angiogenic therapy. The impact of this factor on the therapeutic effect of tumor will undoubtedly profoundly reshape the strategy and direction of anti-tumor therapy in the future [48].

Trans-differentiation of cancer stem cells. The role and mechanism of this model covers the trans-differentiation process of cancer stem cell-like cells into ECs and vascular smooth muscle-like cells, thereby promoting the generation of new blood vessels. This mechanism is also particularly important in the study of cancer angiogenesis [49–53].

Besides the mechanisms, tumors can also induce angiogenesis via lymphangiogenesis and coalescent angiogenesis [39].

**Fig. 2:**
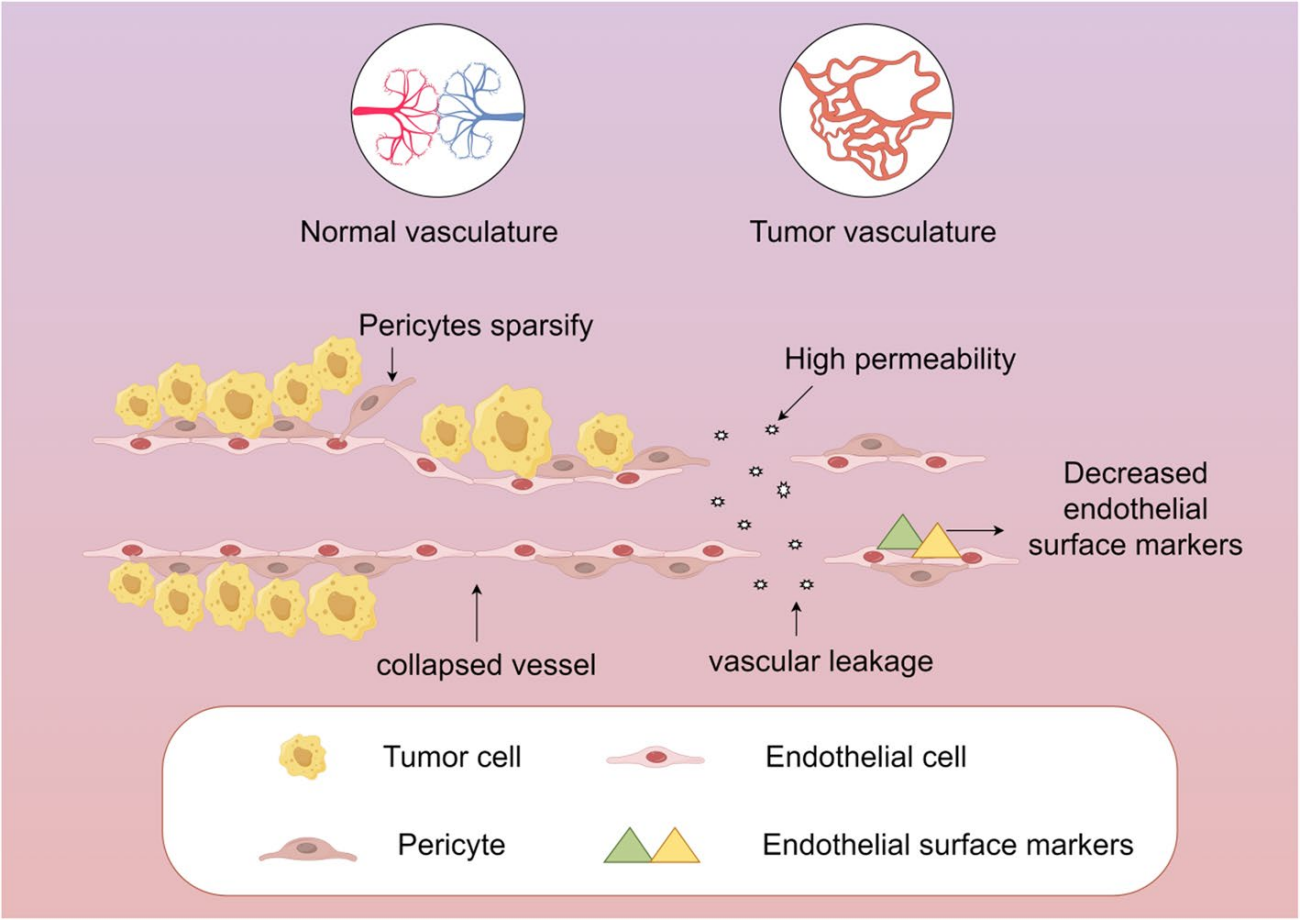
Tumor blood vessels characteristics. Abnormal tumor vascular structure: compared with normal blood vessels, tumor vascular perfusion dysfunction, disordered, distorted shape, and excessive branching; hyperpermeability: an increase in the number of abnormal pores (such as endothelial Spaces, vesicles, and transcellular channels) in the vascular walls of the tumor, widening of connections between endothelial cells, broken or missing basement membranes, and abnormal shapes of endothelial cells that overlap and sometimes protrusion into lumen; Increased vascular leakage: due to the above changes, vascular leakage is aggravated, which disrupts the metabolic balance between the tumor area and the surrounding normal lymphatic system, causes the increase of hydrostatic pressure within the tumor tissue, and aggravates the deterioration of the tumor growth environment; Reduced endothelial surface markers: ECs in tumor vessels may exhibit low levels of surface markers, such as cell adhesion molecules, which may affect the function of the vessel; Pericellular sparsity: Tumor blood vessels may lack pericellular cells, which are critical for maintaining the"resting"state of blood vessels and ensuring proper vascular activity to meet metabolic needs

During the rapid growth of tumor tissue, insufficient nutrient and oxygen supply lead to the abnormal clearance of metabolic wastes, which lead to local hypoxia and acidic environment. This hypoxic state becomes a key factor driving tumor neovascularization. Tumor blood vessels typically exhibit curved and irregular structures with local leakage and compression (Fig. 2) [54, 55]. These abnormalities arise primarily from two factors: (a) abnormal membrane structure—due to dysfunction and reduction of the number of perivascular cells and the disorder of cadherin secretion. These factors together lead to the destruction of the connection between vascular pericytes and basement membrane, thereby reducing the overall stability and integrity of the vessel wall; (b) abnormal lumen structure—uncontrolled tumor cell growth and expansion, constrained by surrounding host tissue, generate mechanical forces that compress intratumoral blood vessels, leading to lumen collapse. In addition, the disordered accumulation of vascular ECs further leads to abnormal luminal structure [7].

Structurally, we found that the tumor vessels showed significant membrane structure and luminal structure abnormalities, which was one of its core features. The disruption of connections between pericytes and the basement membrane results in an incomplete vascular wall, increased permeability, and elevated interstitial fluid pressure, leading to tissue fluid leakage and swelling in and around the tumor tissue [56]. Tumor cells are also prone to invasion and hematogenous metastasis. The collapse of the lumen structure leads to poor perfusion of the blood vessels, insufficient oxygen transport and the inability of metabolites to be transported out in time. The release of VEGF, FGF, TNFα, PlGF, TGF-β, angiopoietin (Ang), etc. [57], and other inflammatory factors not only exacerbates abnormal angiogenesis but also induces immunosuppressive effects [58]. In addition, the low vascular perfusion capacity also leads to the difficulty of drug delivery, which seriously affects the efficiency of tumor treatment [7, 9]. Future research should explore the molecular basis of these biological processes in order to identify and establish potential new therapeutic targets.

## The effect of tumor microenvironment on angiogenesi

The tumor microenvironment (TME) is the key environment for the growth, proliferation and diffusion of tumor cells. TME includes tumor cells, surrounding fibroblasts, immune and inflammatory cells, glial cells, ECM, blood vessels and biomolecules in the adjacent area, which together constitute a complex network and provide key support and promotion for tumor proliferation and survival [59–62]. At present, more and more people realize the importance of TME in tumor biology and begin to realize that tumor and tumor microenvironment are an inseparable whole. The study of TME plays an extremely key role in understanding the mechanism of tumor angiogenesis, growth, invasion and metastasis, as well as promoting the diagnosis, prevention strategies and prognosis evaluation of tumors [61, 63].

The unique phenomena of hypoxia, low PH and interstitial hypertension in TME are rooted in the abnormal structure and function of tumor blood vessels. These conditions promote tumor proliferation, invasion and metastasis [64]. The phenomenon of hypoxia is particularly significant in TME, which is due to the contradiction between the rapid increase in oxygen consumption rate caused by the enhancement of metabolic activity of tumor cells and the relative lag in the ability of new angiogenesis. The adaptive mechanism of tumor cells in the face of hypoxia is mainly regulated by hypoxia-inducible factor- 1 (HIF- 1) [65]. The activation of HIF- 1 increases the expression of pro-angiogenic factors such as VEGF, thereby promoting tumor angiogenesis [66, 67]. Therefore, HIF- 1 is recognized as one of the key targets against tumor angiogenesis.

**Fig. 3:**
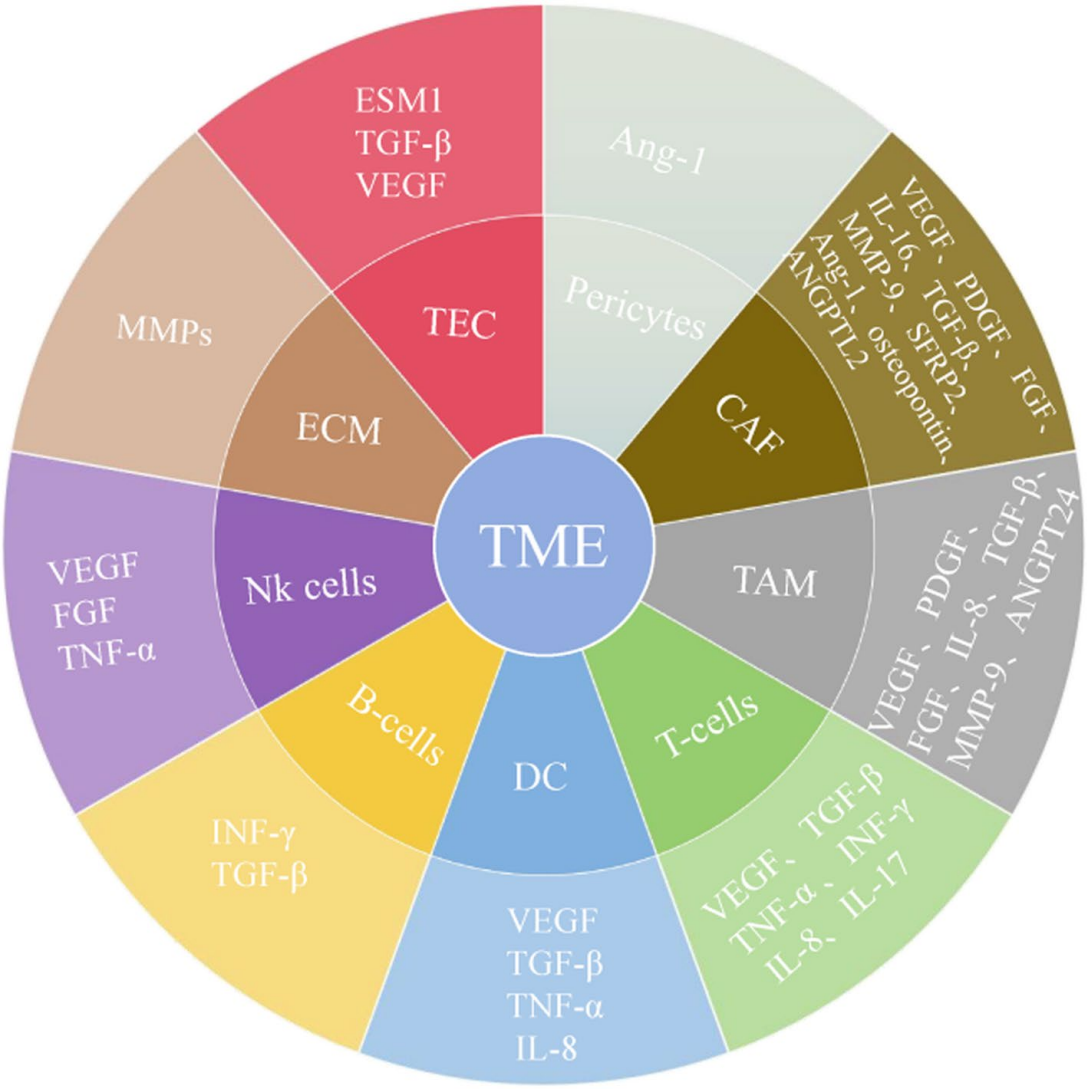
Angiogenesis related factors secreted by various components of the tumor microenvironment

As a dynamic interaction network, the members of TME include diverse cell types, including tumor cells, ECs, fibroblasts, immune cells, and pericytes, as well as a variety of extracellular components including ECM, and key signaling molecules such as cytokines and growth factors [68]. The specific details of the key components are shown below (Fig. 3).

Tumor endothelial cells (TEC). TEC are microscopically distinct from regular EC in that they lack coverage and pericytes are usually separated from ECs, irregular TEC and lack of structural integrity in the vessel wall can lead to leakage [69, 70]. There are significant structural and functional differences between TEC and normal EC. The irregular morphology of TEC and the loss of VE-cadherin together lead to a significant increase in their permeability and disordered cell arrangement. The incomplete and discontinuous basement membrane of tumor blood vessels promotes a further increase in vascular permeability. These structural abnormalities lead to a hypoxic and acidizing environment in the TME region [64]. TEC significantly contributes to tumor angiogenesis and immune cell interactions by secreting factors that influence immune response and promote immunosuppression. In addition, current novel therapies combine anti-angiogenic therapy with immunotherapy, targeting TEC and immune cells, blocking angiogenesis and enhancing effector cell activity within tumors. Clinical trials are exploring these combination therapy strategies and nanotechnology-based approaches for targeting TEC [71].

Pericytes. These cells are a type of parietal cell embedded between the capillary EC and the basement membrane surrounding the postcapillary venules. Its function is to promote the maturation of blood vessels, maintain the normal microcirculation of local tissues and organs, regulate blood flow, vascular permeability and capillary vasomotion, which is an important part of TME [72, 73]. Pericytes support blood vessels structurally and are crucial for angiogenesis and vascular stability. Pericytes and ECs are closely related in anatomical structure, and their interaction is crucial for the occurrence, development, stability, maturation and remodeling of capillaries. The related signaling pathways include PDGF-BB/PDGFR-β, TGFβ1/2, and Ang/Tie- 2, etc. [74]. Through these signaling pathways, signal transduction between pericytes and ECs is enhanced. Therefore, pericytes and related signaling pathways can be used as important targets for anti-tumor angiogenesis, which provides a new direction for anti-tumor angiogenesis therapy.

Cancer-Associated Fibroblasts (CAFs). CAFs play a central role in tumor microenvironment and are the most important type of stromal cells in this environment [75]. The key factors, including VEGF-A, PDGF-C, IL, osteopontin, SFRP2 and FGF- 2, to promote tumor angiogenesis [76]. CAFs play a key role in the process of tumorigenesis, tumor growth and spread through tumor metastasis, angiogenesis, immune evasion and drug resistance. In recent studies, CAFs-derived extracellular vesicles (EVs) affect tumor angiogenesis and lymphangiogenesis by regulating specific miRNAs and other bioactive molecules, thereby promoting tumor growth and metastasis, which provides new insights for the development of targeted therapies against tumor growth and metastasis [77]. Therefore, CAFs have become potential targets for tumor intervention and treatment. A variety of drugs targeting CAFs have been successfully developed and completed the preliminary clinical trials [61].

Immune cells. Immune cells are an important part of TME, and they have pro-or anti-tumor growth effects. Immune cells fall into two categories: adaptive and innate immune cells. One is adaptive immune cells, including T cells, B cells, and natural killer (NK) cells, which are activated in response to encounter with a specific antigen [78]. Once activated, they build immune memory from which to assess potential threats and efficiently mount immune responses to meet challenges. Innate immunity, as a nonspecific defense system that involves macrophages, neutrophils, and dendritic cells (DC) that begin to function within hours after the introduction of foreign antigens [78]. These cells play diverse roles in immune responses, with tumor-associated macrophages (TAMs) being the most prominent in TME [79, 80]. TAMs are frequently linked to cancer progression, poor prognosis, and resistance to treatments, including immunotherapy. They can be classified into the pro-inflammatory M1 subtype, which engulfs and kills cells, or the anti-inflammatory M2 subtype, which aids in wound healing. Both subtypes play roles in tumorigenesis, progression, angiogenesis, and metastasis [81]. TAMs facilitate angiogenic transition by releasing various pro-angiogenic factors [82], such as VEGF [83], PDGF, FGF, IL- 8 [84], TGF-β, and MMP9 [85]. These factors enhance the recruitment and activation of ECs and other cells, promoting vascular network formation [81, 86]. In addition, TAMs promote ECs recruitment and angiogenesis activation by releasing adrenomedullin in melanoma [87].

Extracellular matrix (ECM). As the main non-cellular component in TME, the composition of ECM is complex and diverse, including collagen, galectins, proteoglycans, and glycoproteins [88]. ECM also has important functions in TME. Solid tumors contain a large amount of ECM deposits, accounting for about 60% of the total tumor mass. It not only provides a physical scaffold for tumor epithelial cells and stromal cells, but also is a key factor promoting tumor cell dissemination [89, 90]. The ECM is associated with angiogenic and inflammatory pathways that help promote metastatic TME [89]. Elevated matrix metalloproteinases (MMP) levels in TME regulate ECM homeostasis and facilitate tumor angiogenesis [91]. MMP inhibitors, including incyclinide, have been assessed as potential therapeutic targets for tumor angiogenesis and have demonstrated promising results [91].

The TME is a complex, multifaceted environment with important implications for the angiogenic process. We summarized the angiogenesis related factors secreted by the above components in Fig. 2. Understanding TME component interactions and their secretory factors is crucial for developing effective anti-angiogenic therapies.

## Vascular endothelial cell metabolism and tumor angiogenesi

During development, the vasculature dilates to accommodate and meet the increasing metabolic demands of the body. ECs of the vessel wall is the basic cellular component that constitutes the vascular system and plays a central role in the regulation of angiogenesis. Under normal conditions, mature ECs exhibit extremely high adaptive characteristics and are usually in a quiescent state [92]. However, in the presence of growth factors, they can rapidly transform into a state of activation, proliferation and migration, which mainly depends on the regulation of VEGF signaling [93]. In the process of angiogenesis, ECs are divided into three groups according to their specific location and function: tip cells, stem cells and square cells [93–95]. Angiogenesis, the process by which new blood vessels are derived from existing blood vessels, plays a crucial role in the normal growth and development of an organism, as well as the development of pathological conditions such as cancer [96].

With the deepening of research, it is now believed that the metabolic state of ECs and the stimulation of VEGF are equally important for the biological process of angiogenesis. ECs play a key role in angiogenesis through its diverse metabolic pathways, such as glycolysis, fatty acid oxidation and glutamine metabolism [97]. As the main energy-generating mechanism in ECs, glycolysis is capable of producing up to 85% ATP to provide sufficient power for cellular activities [93, 98, 99]. ECs exhibit a glycolysis rate comparable to many cancer cells and higher than numerous other healthy cell types. After glucose enters the ECs, it is converted to pyruvate via the glycolytic pathway. Under normal physiological conditions, the complete oxidative metabolism of glucose molecules is capable of generating approximately 34 ATP molecules [97]. However, it is worth noting that only a small fraction (less than 1%) of pyruvate produced from the glycolytic pathway is able to further participate in the tricarboxylic acid (thyroid cancerA) cycle to undergo oxidation reactions [93]. In addition, glycolysis produces ATP rapidly, which is essential to ensure cell migration, energy requirements for cytoskeletal remodeling during angiogenesis, and rapid revascularization prior to hypoxic tissue death [99].

ECs of tumor tissue undergoes metabolic remodeling compared to normal ECs [100].TEC are even more dependent than normal ECs on glycolysis to produce ATP, the process being the primary source of ATP production, and exhibit a hyperglycolytic phenotype. Its mechanism of action involves glucose transporter 1 (GLUT1) and the expression of glycolytic activator PFKFB3 [101, 102]. Hypoxia, pro-inflammatory cytokines and hormonal signals in TME act synergistically to up-regulate PFKFB3 expression [99], thereby promoting the switch of oxidative phosphorylation (OXPHOS) to a more efficient glycolytic pathway [92, 101–105]. In contrast to normal EC, TEC activates pentose phosphate and serine biosynthetic pathways to synthesize nucleotides [106]. TEC enhances cell glycolysis by activating cyclooxygenase- 2 (COX2) and upregulating VEGF production [107]. In hypoxic environment, the accumulation of lactate not only affects cellular energy metabolism, but also promotes angiogenesis by activating HIF1α and PI3 K/AKT pathways, which in turn stimulate VEGF signaling [108, 109]. In addition to glycolysis, TEC also carries out metabolic remodeling through other pathways, such as TEC still retains the basic function of mitochondria, OXPHOS can not only increase the cell's ability to use other nutrients to produce energy, but also provide metabolites for biosynthesis to support cell proliferation. Glutamine metabolism in TEC also promotes tumor ECs proliferation [110–112]. Proliferating ECs can also utilize fatty acids to maintain the TCA cycle to support de novo nucleotide synthesis by ECs during proliferation [113].At present, it has been proved that tumor angiogenesis can be inhibited by inhibiting PFKFB3 and inhibiting fatty acid synthesis [106]. Further exploration of metabolic changes in the TME and a deeper understanding of ECs metabolism are crucial to guide the development of targeted tumor growth inhibition and angiogenesis drugs.

ECs metabolic pathways are considered to be key mechanisms regulating angiogenesis, and this finding provides potential targets for exploring and developing anti-angiogenic therapies. Recent studies have shown that the metabolic state of ECs and the stimulatory effect of VEGF play a key role in the core mechanism of the biological process of angiogenesis. [99, 114]. Identifying and understanding the molecular and metabolic heterogeneity between normal and TEC is the key to designing more targeted and effective therapeutic strategies. Exploring the similarities and differences of ECs metabolism in normal and TME is expected to open up a new perspective for the development of anti-angiogenic drugs and the optimization of treatment strategies for vascular diseases.

## Molecular mechanisms and signaling pathways in tumor angiogenesis

Tumor angiogenesis is influenced by intricate interactions between molecular mechanisms and signaling pathways. A variety of angiogenic factors and their receptors can accelerate the formation of tumor-associated blood vessels. A variety of pro-angiogenic factors have been found to be involved in this process. These include growth factors (VEGF, FGF, PDGF, EGF, etc.), adhesion factors (integrin, cadherin), proteases (MMP), angiopoietins, endothelial cell specific molecule- 1 (ESM1), ANGPTLs, apelin (APLN) and chemokines. These factors participate in the regulation of tumor neovascularization by activating a variety of signal transduction pathways (Fig. 5a). At multiple stages of tumor angiogenesis, these factors are usually expressed concurrently and collaborate efficiently at various stages of tumor angiogenesis [36]. We will briefly discuss these pro-angiogenic factors and their signaling pathways and highlight their key roles in anti-cancer angiogenesi

### Growth factors and growth factor receptors

VEGF/VEGFRs. VEGF plays a key role in regulating the permeability and angiogenesis of blood vessels, especially in tumor-associated angiogenesis. The VEGF family is composed of the following members: VEGF-A/B/C/D, PlGF, and VEGF-E and svVEGF, which are encoded by non-human genomes [115, 116]. The tyrosine kinase receptor VEGFR consists of three major components: a domain that spans the cell membrane, an extracellular ligand-binding domain with an Ig-like structure, and an intracellular tyrosine kinase domain [117]. Members of the VEGF family fulfill their biological functions by binding to VEGFR. VEGF-A/VEGFR- 2 is considered to be the core signaling pathway of angiogenesis in physiological and pathological conditions. VEGF-A mediates the activation of downstream signaling pathways such as PI3 K/AKT/mTOR, p38 MAPK, Ras/Raf/MEK/ERK by VEGFR- 2. These signaling pathways not only promote ECs growth and survival, but also promote angiogenesis [118, 119]. Research indicates that VEGFR- 2 overexpression is present in solid tumors like melanoma and ovarian cancer (OC) [120, 121], which makes VEGF-A/VEGFR- 2 a key target for angiogenesis inhibitors [122].

PDGF/PDGFRs. The PDGF family consists of PDGF-A/B/C/D, which ensure the stability of neovascularization by promoting the maturation of blood vessels and the recruitment of pericytes through two cell surface tyrosine kinase receptors, PDGFR-α and PDGFR-β [36]. Therefore, therapies targeting the PDGF/PDGFR signaling pathway are also considered to be powerful means against tumor angiogenesis.

EGF/EGFRs. EGF is a single-stranded polypeptide consisting of 53 amino acids. EGFR is composed of four proteins: EGFR (ErbB- 1 HER1), ErbB- 2 (HER2), ErbB- 3 (HER3), and ErbB- 4 (HER4) [123]. In healthy tissues, EGFR plays a regulatory role in cell growth and differentiation. However, in the tumor body, due to its abnormal expression or activity, the tumor takes on aggressive characteristics and further accelerates the formation and development of tumor blood vessels [124–126]. EGF/EGFRs directly stimulate the formation of tumor blood vessels by regulating the expression of genes, thereby further accelerating the invasion and spread of tumors, and are associated with many types of cancers such as breast cancer (BC) and OC [126, 127]. Because it is involved in the growth of cells and the formation of new blood vessels, it is often associated with poor prognosis.

FGF/FGFRs. FGF, which is composed of 23 structurally diverse proteins [128, 129], plays a key role in wound healing and is the first growth factor identified to be closely related to angiogenesis [10]. FGF- 2, namely basic fibroblast growth factor (bFGF), plays a central role in maintaining its physiological function and promoting the development of tumors [130, 131]. As an important angiogenic factor, FGF- 2 plays an important role. It not only promotes angiogenesis by inducing the secretion of MMP, plasminogen activator and collagenase, but also participates in the degradation of extracellular matrix and tissue remodeling [132]. FGFR, a transmembrane receptor family, promotes angiogenesis in humans by self-phosphorylation and activating SrC-family kinases, PLCγ/DAG/PKC, Ras/Raf-MAPK, and PI3 K/AKT pathways [133–135].

HGF/c-Met. Hepatocyte growth factor (HGF) not only plays a key role in promoting the proliferation and differentiation of normal cells as a multifunctional factor [136], but also has attracted much attention because it can significantly enhance the invasion and metastasis of tumor cells [137]. c-met is a specific receptor for HGF [138, 139]. HGF/c-Met signaling pathway plays a central role in wound healing, tissue regeneration, and embryogenesis [140–142]. The abnormal HGF/c-Met signaling pathway, involving the amplification of c-Met gene, secondary mutation, transcriptional regulation imbalance and the abnormal HGF autocrine or paracrine caused by the overexpression of c-Met, can significantly promote the proliferation, invasion, angiogenesis [143, 144], drug resistance and ultimately affect the prognosis of patients [140, 145–147]. Due to the critical role of HGF/c-Met axis in angiogenesis and anti-tumor drug resistance in pathophysiological processes, this pathway has gradually become an attractive target for anti-tumor angiogenesis therapy.

IGF/IGFRs. Insulin-like growth factor (IGF) is a polypeptide molecule that plays a key role in the regulation of human growth, development and energy metabolism [148]. It includes three key ligands: insulin, IGF1 and IGF2—that engage in the physiological cycle via autocrine, paracrine, and endocrine mechanisms [149]. IGF and IGFRs play a central role in the process of cell growth, proliferation and differentiation. IGF plays a key role in promoting angiogenesis by enhancing the viability and migration of ECs. Specifically [150], IGF1 and IGF2 can stimulate the synthesis of VEGF, which promotes the process of angiogenesis.

TGF-β. Transforming growth factor-β (TGF-β), a key signaling molecule, not only plays a central role in maintaining homeostasis, but also actively participates in tissue repair processes and regulates immune responses [151]. The interaction between TβRI, TβRII and TβRIII significantly regulates cell growth, differentiation and apoptosis [152]. In the development of the tumor, this factor plays a two-sided role: in the initial stage, its main function is to curb the proliferation of the tumor; However, when a tumor reaches the mature stage, it promotes tumor growth by promoting processes such as epithelial-mesenchymal transition (EMT) and angiogenesis [153–155]. TGF-β is closely related to a variety of tumors, and it may become a key therapeutic target to prevent tumor angiogenesis and overcome drug tolerance.

In the development of the tumor, this factor plays a two-sided role: in the initial stage, its main function is to curb the proliferation of the tumor.

### Transcription factors

HIF- 1. Hypoxia plays a central role in the tumor microenvironment. It not only affects the drug resistance of tumors, but also is closely related to tumor angiogenesis [156]. HIF- 1, a regulator that plays a central role in cell survival and metabolism, is composed of two subunits, HIF- 1α and HIF- 1β, forming a heterodimer structure. HIF- 1α undergoes hydroxylation and degradation under sufficient oxygen. However, in response to hypoxia, HIF- 1α binds to HIF- 1β to form a complex that activates the expression of key pro-angiogenic genes such as VEGF [157]. HIF- 1α is not only closely related to the progression of tumors, but also affects the prognosis of patients [158]. Therefore, it has shown great clinical application prospects as a potential target in anti-tumor treatment strategies.

NF-κB. Nuclear factor-κB (NF-κB) is a key intracellular transcription factor that plays a central role in many biological processes such as inflammatory response, immune regulation, cell growth and survival, and promotion of angiogenesis [159–161]. Activation of NF-κB usually involves a complex set of signaling pathways, including phosphorylation of IκB, a protein that inhibits NF-κB, and the subsequent transfer of NF-κB from the cytoplasm to the nucleus [162]. The abnormal activation of NF-κB is related to the occurrence and development of many diseases, such as autoimmune diseases and cancer [163]. In the pathological process of cancer, it plays an important indirect role in the occurrence, development and metastasis of many types of cancer by regulating the expression of angiogenic factors, especially VEGF. [164]. Inhibiting NF-κB signaling diminishes tumor angiogenesis, presenting a viable anti-angiogenic strategy.

### Epigenetic regulation

Epigenetic regulation drives tumor angiogenesis through mechanisms such as DNA methylation, RNA methylation (m6 A), histone modification and non-coding RNA[165–168]. DNA methylation affects angiogenesis by regulating the expression of VEGF, IL- 8 and other genes. For example, DNMT3B-mediated hypermethylation of miR- 200 family enhances TGF-β1 signaling pathway and promotes CAFs to secrete pro-angiogenic factors [169]. The m6 A enhances the proangiogenic phenotype of CAFs by modifying genes such as COL10 A1 [170, 171]. Histone modifications regulate angiogenesis-related genes through chromatin remodeling, such as HDAC inhibitor entinostat up-regulates anti-angiogenic genes SERPINF1 and THBS2 and inhibits vascular mimicry [172]. Non-coding Rnas, such as miR- 21, promote angiogenesis by targeting STAT3 and other pathways [173]. Currently, lncRNAs and circRNAs have been identified as novel and multifunctional players involved in tumor angiogenesis through manipulation of angiogenic factors [174]. Several well-known lncRNAs, such as H19 and MALAT1, have been identified as important regulators of angiogenic factors [175]. Similarly, circRNA and circRhoC enhanced OC angiogenesis by regulating VEGFA expression [176]. Epigenetic drugs combined with immune checkpoint inhibitors are becoming a new strategy for anti-angiogenesis treatment.

### Other angiogenic factors

**Fig. 4:**
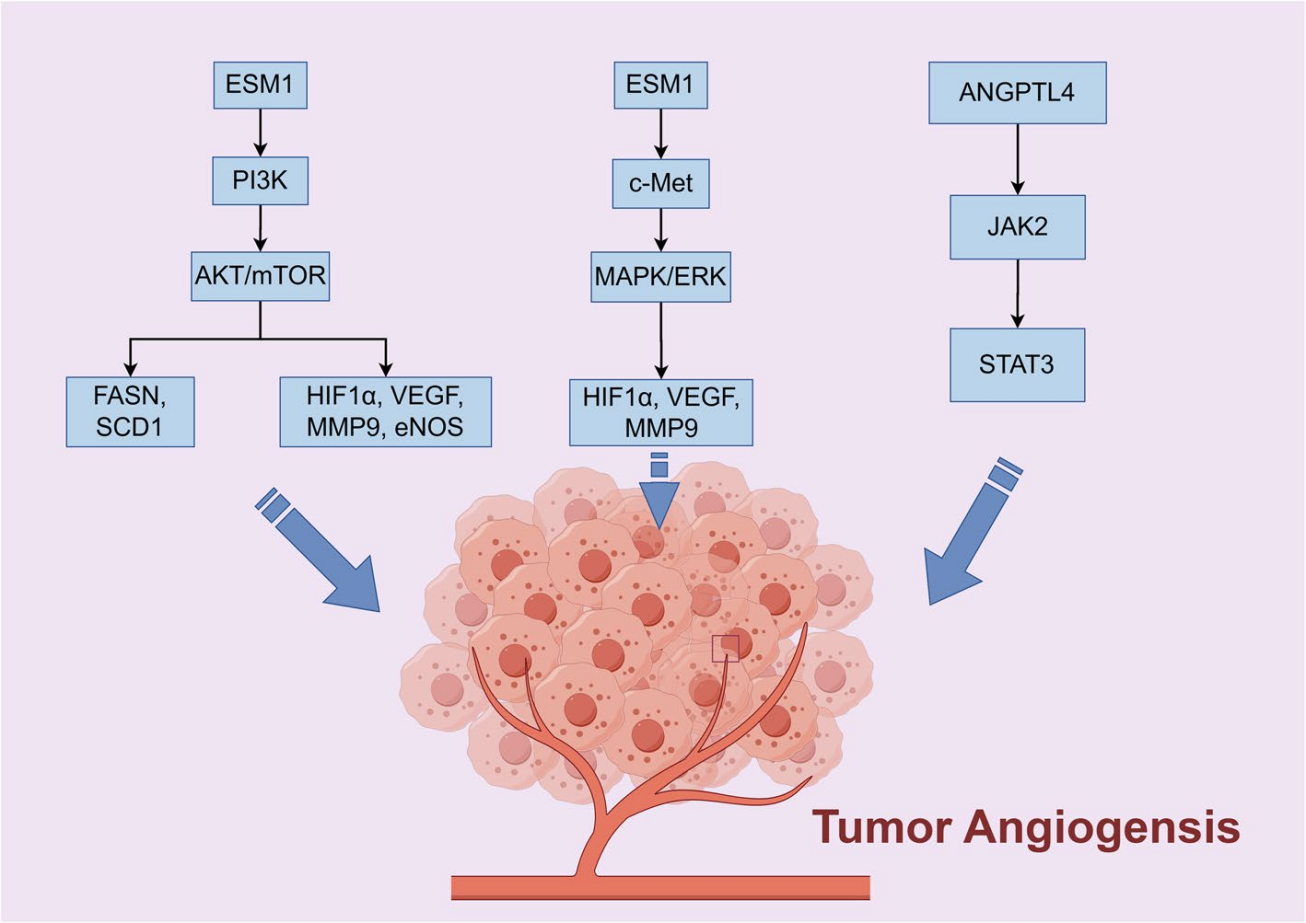
ESM1 and ANGPTL4 promote tumor angiogenesis signaling pathways

ESM1. Endothelial cell specific molecule 1 (ESM1), primarily expressed in human lung and kidney ECs, is crucial for angiogenesis and serves as a significant clinical marker for various tumors [177]. Physiologically present in proliferative tissues, it can also be ectopically expressed in various cancers, including bladder cancer, BC, OC, colorectal adenocarcinoma (COAD), gastric adenocarcinoma (STAD), lung adenocarcinoma (LUAD), and thyroid cancer (THCA) [178]. It influences cancer progression through proliferation, migration, invasion, and drug resistance [179, 180]. ESM1 regulates critical signaling pathways in cancer development, including the AKT/NF-κB/Cyclin D1, PI3 K/Akt/mTOR, Wnt/β-catenin, DLL4-Notch, AKT/eNOS, and NF-κB/iNOS pathways [178]. ESM1 expression is regulated by TNF-α, IL- 1β, HIF, and VEGF. Our research indicates that ESM1 is pivotal in cancer metabolic reprogramming. We found that ESM1 can interfere with glucose metabolism and fatty acid synthesis. In LUAD, ESM1 up-regulates SCD1 and FASN through the Akt signaling pathway to promote lipid metabolic reprogramming and accelerate angiogenesis (Fig. 4a) [181].SCD1 and FASN are crucial lipid-metabolizing enzymes that facilitate fatty acid synthesis [182]. ESM1 promotes OC cell proliferation and angiogenesis by up-regulating the PI3 K/Akt pathway (Fig. 4b) [178]. A recent study has shown that ESM1 is significantly associated with gastric cancer peritoneal metastasis (GCPM) and plays an important role in promoting angiogenesis. Specifically, ESM1 upregulates the expression of VEGF-A, HIF1α and MMP9 by binding to c-Met and activating the MAPK/ERK pathway (Fig. 4c) [183], which play key roles in tumor angiogenesis, invasion and metastasis. These findings not only reveal the important role of ESM1 in cancer progression, but also provide new clues for ESM1 and its regulated signaling pathways to become therapeutic targets in the future.

ANGPT2-TIE2 signaling pathway is a key system to regulate angiogenesis and homeostasis, which is composed of angiopoietins (ANGPT1/ANGPT2) and TIE receptors (TIE1/TIE2) [184, 185]. As an endothelial cell-specific receptor tyrosine kinase, TIE2 mediates vascular development, maturation and microenvironment stability through ligand binding [186]. ANG1 activates the phosphorylation of TIE2 to promote vascular stability. ANG2 competitively inhibits the binding of ANG1 to TIE2, blocks its phosphorylation and weakens vascular integrity, while enhancing the sensitivity of endothelial cells to VEGF and synergistically promoting angiogenesis. VE-PTP further regulates this pathway activity by dephosphorylating TIE2. Studies have shown that ANG2 plays a central role in the abnormal proliferation of tumor blood vessels, and its high expression is closely related to tumor progression, which can not only drive the sprouting of new blood vessels, but also accelerate tumor metastasis by destroying vascular homeostasis [187]. Therefore, targeting ANG-TIE2 pathway has become an important direction of anti-tumor therapy. At present, drugs targeting ANG2 single target, ANG2/VEGF dual target, ANG1/ANG2 bifuncular antibody and TIE2/VE-PTP inhibitor have entered the clinical development stage. In addition, the potential of ANG2 as a prognostic marker and a potential therapeutic target has attracted much attention, while TIE2 targeted drugs are in the early stages of investigation. Due to its dual role in vascular remodeling and tumor microenvironment regulation, ANG2-TIE2 axis has become a research hotspot in tumor immunotherapy and anti-angiogenesis combination therapy [188].

ANGPTLs. ANGPTLs share structural similarities with Ang [189]. ANGPTLs do not interact with the angiopoietin receptors Tie2 or Tie1 [190], yet they influence angiogenesis and participate in metabolic processes and inflammation related to cancer progression [191]. Previous studies have revealed that ESM1 not only significantly enhances the proliferation of OC cells by activating the Akt signaling pathway, but also acts synergistically with ANGPTL4 to further promote its migration, invasion, angiogenesis, and lipid metabolism reprogramming process, thereby providing a key support mechanism for the development of OC [181, 192]. ANGPTL4 plays a role in pathophysiological processes, including metabolic reprogramming, angiogenesis, proliferation, and metastasis. Research shows that ANGPTL4 was significantly increased in patients with GC. The contradictory effects of ANGPTL4 on proliferation, migration, invasion and angiogenesis in multiple cancer cell lines, including AGS, MKN7 and SNU5 [193]. At present, the understanding of ANGPTLs signaling pathway is not comprehensive, and there are many unsolved mysteries. The potential role and mechanism of this pathway in cancer progression needs to be further explored and revealed.

The molecular mechanisms and signaling pathways regulating tumor angiogenesis are extremely complex and interrelated, which mainly drive the process of tumor angiogenesis by enhancing the proliferation ability of target cells, enhancing their survival advantages, promoting migration and changing cell morphology. In addition to the signaling pathways and biomolecules described above, angiopoietin/Tie [184], Notch-Delta/Jagged [194], Ephrins/EphR [195], Apelin/APLNR [196], Slit/Robo pathways [197], as well as adrenomedullin [198], COX- 2 [199], CXC chemokine [200], interleukin [201], interferon [202], integrin [203], nitric oxide synthase (NOS) [204], and polytropic cell protein (PTN) [205], steroid hormones [206], MMP [207], thrombospondin (TSP), secreted protein acidic and rich in cysteine (SPARC) [208–210], Fibronectin 1 (FN1) [211, 212], and programmed death ligand 1 (PD-L1) also play a role in tumor angiogenesis [10, 213, 214]. Understanding these pathways and molecules will be critical to advancing targeted therapies to disrupt tumor angiogenic processes. Future research should aim to elucidate pathway interactions and identify novel therapeutic targets.

## Anti-angiogenic targeted therapies in oncology: classification, clinical efficacy, and emerging challenges

Anti-angiogenic therapy, a key strategy to effectively curb tumor growth and metastasis by blocking angiogenesis, has become one of the important means in the field of malignant tumor treatment. Although a number of studies have clearly pointed out that tumor angiogenesis is affected by a variety of regulatory factors, the current development and clinical application of anti-angiogenesis inhibitors are still highly focused on the strategy of targeting the signal transduction pathway mediated by VEGF and its receptor VEGFRs [215]. This not only reflects the central role of VEGF/VEGFRs signaling pathway in the process of tumor angiogenesis [216], but also reflects the limitations and challenges of existing therapeutic strategies. With the in-depth exploration of other potential regulatory mechanisms, more comprehensive and efficient anti-angiogenic therapies may be developed in the future to more precisely block the vascular support required for tumor growth. At present, the US Food and Drug Administration (FDA) has approved 14 targeted Anti-angiogenic drugs [11], mainly including monoclonal antibodies and tyrosine kinase inhibitors, which have shown significant effects in the treatment of a variety of cancers and other human diseases [10].

Table 1. provides a systematic classification of anti-angiogenic drugs, including targets and indications; Table 2 compares the efficacy and safety data of various classes of anti-angiogenic therapies from clinical trials.

**Table 1:** Classification, molecular targets, and tumor applications of anti-angiogenic agents in oncology

Classification of drugs	drugs	Targets	Tumor type	Reference
Monoclonal antibodies	Bevacizumab (Avastin)	VEGF-A	colorectal cancer, NSCLC, RCC, GBM, CC, metastatic BC	[63, 217, 218]
	Ramucirumab (Cyramza)	VEGFR- 2	NSCLC, Advanced GC, GEJ adenocarcinoma, metastatic colorectal cancer, metastatic urinary tract epithelial cancer	[219, 220]
	Olaratumab (Lartruvo)	PDGFR-α	soft tissue sarcoma	[221]
	Bevacizumab-awwb (Mvasi)	VEGF	colorectal cancer, NSCLC, RCC, GBM, CC	[222, 223]
	Durvalumab	PD-L1	OC, TNBC, Endometrial Carcinoma,HCC	[224]
	Atezolizumab	PD-L1	Metastatic nonsquamous NSCLC, Advanced TNBC, HCC	[225]
Recombinant fusion proteins	Aflibercept (Eylea)	VEGF-A/B, PlGF	colorectal cancer	[226]
	ziv-Aflibercept (Zaltrap)	VEGF-A/B, PlGF	colorectal cancer	[227]
HIF- 1 inhibitor	EZN- 2208	HIF- 1	Metastatic colorectal cancer,	[228]
	CRLX101	HIF- 1α	RCC, PRAD	[229, 230]
	2ME2	HIF- 1α	RCC	[231]
	17-AAG	HIF- 1α	Melanoma	[232]
Classification of drugs	drugs	Targets	Tumor type	Reference
	Vorinostat	HIF- 1α	HNSCC	[232]
	PT2977	HIF- 2α	RCC	[233]
mTOR inhibitors	Temsirolimus (Torisel)	mTOR	RCC	[234]
	*Everolimus (Afinitor)*	mTOR	RCC, SEGA, pNET, HER2- BC	[235]
Immunomodulatory agents	Thalidomide (Thalomid)	VEGF-A, NF-κB, TNF	multiple myeloma	[236]
	Lenalidomide (Revlimid)	VEGF-A, NF-Κb, TNF	multiple myeloma, mantle cell lymphoma	[237, 238]
Classification of drugs	drugs	Targets	Tumor type	Reference
Tyrosine kinase inhibitors (RTKI)	Sorafenib (Nexavar)	VEGFR- 1/2/3, PDGFR-β, Raf, Ret, BRAF, c-Kit, Flt- 3	RCC, hepatocellular carcinoma, thyroid cancer, differentiated thyroid cancer	[239, 240]
	Sunitinib (Sutent)	VEGFR- 1/2/3, PDGFR-α/β, c-Kit, CSF- 1R, Ret, Flt- 3	RCC, gastrointestinal stromal tumor, pNET	[241, 242]
	Pazopanib (Votrient)	VEGFR- 1/2/3, c-Kit, PDGFR-α/β,	RCC, soft tissue sarcoma	[243, 244]
	Vandetanib (Caprelsa)	VEGFR- 2/3, EGFR, Ret	medullary thyroid cancer	[245, 246]
	Regorafenib (Stivarga)	VEGFR- 1/2/3, PDGFR-β, FGFR- 1, c-Kit, Tie- 2, BRAF, Raf- 1, Ret	colorectal cancer, hepatocellular carcinoma, gastrointestinal stromal tumor	[247, 248]
Classification of drugs	drugs	Targets	Tumor type	Reference
	Axitinib (Inlyta)	VEGFR- 1/2/3, PDGFR-α/β, c-Kit	RCC	[249, 250]
	Ponatinib (Iclusig)	VEGFRs, FGFRs, PDGFRs, EPHs, Ret, LYN, ABL, Src, LCK, BLK, c-Kit, FGR, HCK, FRK, FYN, c-FMS,	Ph+ AML, CML	[251]
	Cabozantinib (Cometriq)	VEGFR- 2, c-Kit, c-Met, Flt- 3, RON, Ret, AXL, Tie- 2	medullary thyroid cancer, hepatocellular carcinoma, RCC,	[252]
	Apatinib (Aitan)	VEGFR- 2, c-Kit, Src	GC	[253]
Classification of drugs	drugs	Targets	Tumor type	Reference
	Nintedanib (Ofev)	VEGFRs, PDGFRs, FGFRs, LCK, Flt- 3, Src, LYN	NSCLC	[254]
	Vorolanib (CM082)	VEGFRs, PDGFR	advanced RCC, lung cancer	[21]
	Lenvatinib (Lenvima)	VEGFRs, PDGFRs, FGFRs, c-Kit, Ret	differentiated thyroid cancer, thyroid cancer, hepatocellular carcinoma, RCC, Endometrial Carcinoma	[255–257]

*GBM* Glioblastoma, *CC* Cervical cancer, *BC* Breast cancer, *GC* Gastric cancer, *GEJ* Gastroesophageal junction, *TNBC* Triple-negative breast cancer, *PRAD* Prostate adenocarcinoma, *HNSCC* Head and neck squamous cell carcinoma, *SEGA* Subependymal giant cell astrocytoma, *HER2* Human epidermal growth factor receptor 2, *pNET* Pancreas neuroendocrine tumor, *Ph* + *AML* Philadelphia chromosome-positive acute myeloid leukemia, *CML* Chronic myeloid leukemia, *NSCLC* Non-small cell lung cancer, *RCC* Renal cell carcinoma, *HCC* Hepatocellular carcinoma

**Table 2:** Comparative efficacy of anti-angiogenic drugs by mechanism and clinical outcomes in tumors

Agents	Targets	Cancer Types	Study Phase	Efficacy Outcomes	Adverse Events	Trial Identifier
Niraparib + Bevacizumab	PARP, VEGF-A	Epithelial OC	II	PFS: at 18 months was 62% (95% CI: 51.9% to 71.2%)	Hypertension: 57/105 (54.29%); Anaemia: 56/105 (53.33%); Thrombocytopenia: 37/105 (35.24%)	NCT03326193
Ramucirumab + Docetaxel	VEGFR- 2	Previously treated metastatic NSCLC	III	OS: 10.5 vs. 9.1 months (HR 0.86); PFS: 4.5 vs. 3.0 months (HR 0.76); ORR: 22.9% vs. 13.6%	Febrile neutropenia: 86/627 (13.72%); Diarrhoea: 13/627 (2.07%); Stomatitis: 14/627 (2.23%)	NCT01168973
Olaratumab + Nabpaclitaxel + Gemcitabine	PDGFR-α	Metastatic Pancreatic Ductal Adenocarcinoma	Ib/II	OS:9.1 vs. 10.8 months (HR = 1.05); PFS: 5.5 vs. 6.4 months (HR = 1.19); ORR: 30.5% vs. 33.8%	Fatigue: 53/81 (65.43%); Anemia: 46/81 (56.79%); Neutropenia: 20/81 (24.69%); Thrombocytopenia: 7/81 (8.64%)	NCT03086369
Durvalumab + Bevacizumab + TACE	PD-L1, VEGF-A	Unresectable Hepatocellular Carcinoma	III	PFS: 27.9 months (95% CI: 27.4% to 30.4%)	Hypertension: 9/154 (6%)	NCT03778957
Atezolizumab + Bevacizumab	PD-L1, VEGF-A	Unresectable Hepatocellular Carcinoma	III	OS: 19.22 vs. 13.40 months; PFS: 6.8 vs 4.3 months; ORR: 27.3% vs 11.9%;	Hypertension: 118/329 (35.87%); Fatigue: 76/329 (23.10%); Gastrointestinal hemorrhage: 3/329 (0.9%)	NCT03434379
Aflibercept + FOLFIRI	VEGF-A/B, PIGF	Metastatic Colorectal Cancer,	II	PFS: at 6 months was 58.8% (90% CI: 45.7% to 72.0%); Median PFS: 7.3 months (95% CI: 5.5 to 11.0 months); Median OS: 18.8 months (95% CI: 12.9 to 26.6 months); ORR: 20.9% (95% CI: 10.0% to 36.0%)	Neutropenia: 55.8% (Grade ≥ 3); Leukopenia: 25.6% (Grade ≥ 3); Febrile Neutropenia: 11.6% (Grade ≥ 3); Fatigue: 9.3% (Grade ≥ 3)	JRCTs011190006
Agents	Targets	Cancer Types	Study Phase	Efficacy Outcomes	Adverse Events	Trial Identifier
Ziv-aflibercept + Pembrolizumab	VEGF-A/B, PD- 1	Advanced Melanoma	I	PR: 20% (2/10); SD: 20% (2/10); OS: 13.2 months (90% CI: 7.3 to 20.4 months); PFS: 3.0 months (90% CI: 2.6 to 8.2 months)	Hypertension: 80%; Headache: 50%; Fatigue: 50%; Encephalitis: 10%; Meningitis: 10%	NCT02298959
CRLX101 + Bevacizumab	HIF1α, HIF2α, VEGF	Advanced RCC	II	PFS: 3.7 months (95% CI: 2.0 to 4.3); ORR: 4.8% vs. 14%; OS: 16.1 months vs. 16.4 months	Fatigue: 41.8%; Nausea: 36.4%; Hypertension: 7.3%; Anemia: 20.0%	NCT02187302
Bevacizumab + Temsirolimus + Valproic Acid	VEGF-A, mTOR, HDAC	Advanced solid tumors (including head and neck squamous cell carcinoma, OC, BC, etc.)	I	ORR: 7.9%; CBR (PR + SD ≥ 6 months): 21%; Median treatment cycles: 2 (range 1–22)	Lymphopenia (14.9%); Thrombocytopenia (8.5%); Mucositis (6.4%); Hyperlipidemia (5.3%); Bowel perforation (4.3%); CNS ischemia (2.1%)	NCT01552434
Sorafenib + HAIC	VEGF, PDGFR, Raf	Hepatocellular Carcinoma	III	OS: 13.5 vs. 7.5 months; PFS: 7.0 vs. 2.5 months	Abdominal pain (79.8%); ALT increased (38.6%); AST increased (49.7%); Thrombocytopenia (27.8%); Nausea (30.9%); Vomiting (9.9%); Diarrhea (19.1%); Rash (28.4%); Hypertension (23.5%); Fatigue (9.6%)	NCT02774187
Cabozantinib + Atezolizumab	VEGFR2, MET, AXL, PD-L1	Hepatocellular Carcinoma	III	OS: 16.5 vs. 15.5 months; PFS: 6.9 vs. 4.3 months	Hypertension (9%); Palmar-plantar erythrodysaesthesia (8%); Aspartate aminotransferase increased (10%)	NCT03755791
Agents	Targets	Cancer Types	Study Phase	Efficacy Outcomes	Adverse Events	Trial Identifier
Vorolanib + Everolimus	VEGFR2, PDGFR-β, RET, SCFR, mTOR	Metastatic RCC	III	PFS: 10.0 months (95% CI: 8.2 to 10.4); ORR: 24.8%; OS: 30.4 months (95% CI: 16.5 to NE)	Proteinuria (57.9%); Anemia (57.9%); Leukopenia (55.6%); Thrombocytopenia (50.4%); Hypertension (18.8%); Diarrhea (39.8%); Oral Ulcer (33.1%); Facial Edema (32.3%)	NCT03095040
Lenvatinib + Pembrolizumab	VEGFR1 - 3, PDGFR-α, c-Kit, RET	Squamous Cell Carcinoma of the Head and Neck	Ib/II	ORR: 40.9% (95% CI: 20.7 to 63.6); PFS: 4.4 months (95% CI: 4.0 to 9.8)	Cardiac failure congestive 1/22 (4.55%); Supraventricular tachycardia 1/22 (4.55%); Fatigue1/22 (4.55%)	NCT02501096

*PFS* Progression-free survival, *OS* Overall survival, *HR* Hazard ratio, *ORR* Objective response rate, *TACE* Transarterial chemoembolization, *PR* Partial Response rate, *SD* Stable disease rate, *OS* Overall survival, *CBR* Clinical benefit state, *HAIC* Hepatic arterial infusion chemotherapy, *NE* Not estimable

### Monoclonal antibodies

Bevacizumab (Avastin®), an anti-VEGF monoclonal antibody, has demonstrated therapeutic effectiveness across multiple malignancies (Table 1.) [63]. A phase III clinical trial enrolling 878 individuals with recurrent or advanced non-small cell lung cancer (NSCLC) revealed that combining bevacizumab with paclitaxel and carboplatin chemotherapy resulted in superior outcomes compared to chemotherapy alone. Specifically, the regimen enhanced median overall survival (OS) (12.3 vs. 10.3 months), progression-free survival (PFS), and objective response rates (ORR). However, adverse events such as significant bleeding (4.4% vs. 0.7%) and treatment-related fatalities (15 vs. 2 cases) were more frequent in the bevacizumab group. These findings supported FDA approval of bevacizumab combined with paclitaxel and carboplatin for NSCLC management [258]. This study highlights the significant survival benefit of bevacizumab in selected NSCLC patients, while also pointing out the increased risk of serious side effects. On May 29, 2020, the FDA approved the PD-L1 monoclonal antibody atezolizumab combined with bevacizumab as first-line therapy for unresectable or metastatic hepatocellular carcinoma (HCC) in systemic treatment-naïve patients. This combination remains the sole immunotherapy regimen approved by the FDA for initial HCC management [259]. Additionally, bevacizumab-awwb is a bevacizumab biosimilar with the same mechanism of action as the originator. Its clinical equivalence was confirmed in the MAPLE phase III trial, which reported comparable efficacy and safety profiles to the originator in advanced NSCLC cohorts [260].

Ramucirumab (Cyramza®), a fully human IgG1 monoclonal antibody directed at the extracellular domain of VEGFR- 2, inhibits tumor angiogenesis by blocking VEGF signaling (Table 1.) [261]. Its therapeutic utility has been corroborated across clinical studies. For instance, in the phase III REVEL trial involving NSCLC patients refractory to platinum-based chemotherapy, the addition of ramucirumab to docetaxel enhanced OS (10.5 vs. placebo 9.1 months) and PFS (4.5 vs. 3.0 months). Similarly, the phase III RAINBOW trial demonstrated further improvements in OS (9.6 vs. 7.4 months) and PFS (4.4 vs. 2.86 months) when ramucirumab was combined with paclitaxel, leading to its FDA approval in 2014 for GEJ adenocarcinoma following prior therapy [262]. Additionally, a phase II trial (NCT02898077) indicated that ramucirumab-paclitaxel combination therapy exhibited both safety and efficacy in second-line management of advanced GC or GEJ adenocarcinoma.

Olaratumab (Table 1.), a monoclonal antibody targeting PDGFRα, is used to treat soft tissue sarcomas [221]. A randomized, double-blind phase Ib/II study evaluating olaratumab combined with albumin-bound paclitaxel and gemcitabine failed to demonstrate significant improvements in median OS (9.1 vs. 10.8 months) or PFS (5.5 vs. 6.4 months) compared to placebo in metastatic pancreatic ductal adenocarcinoma [263]. Although its safety profile was consistent with that in previous studies, this combination therapy did not meet the prespecified primary end point, which suggests that its use in metastatic pancreatic ductal adenocarcinoma needs further exploration.

Durvalumab and Atezolizumab are both anti-PD-L1 monoclonal antibodies, which can enhance T cell immune response to tumors by blocking the binding of PD-L1 to PD- 1 (Table 1.) [224]. In the treatment of hepatocellular carcinoma, Durvalumab combined with bevacizumab showed significant efficacy in the HIMALAYA phase III trial, with a median OS of 16.43 months and ORR of 20.1%, significantly better than sorafenib (OS of 13.77 months and ORR of 5.1%) [264]. Durvalumab has also shown potential when paired with locoregional therapies such as transarterial chemoembolization (TACE), stereotactic body radiotherapy (SBRT), and transarterial radioembolization (TARE). The IMbrave150 phase III trial evaluated atezolizumab plus bevacizumab as first-line therapy for unresectable HCC. This regimen significantly extended median OS (19.2 vs. 13.4 months) and PFS (6.8 vs. 4.3 months) compared to sorafenib, alongside a higher ORR (27.3% vs. 11.9%). These results led to FDA approval in 2020, establishing this combination as a standard frontline treatment [265]. Together, these agents broaden HCC therapeutic options by synergistically combining immune checkpoint inhibition and anti-angiogenic effects.

### Recombinant fusion proteins

Aflibercept and ziv-aflibercept are recombinant fusion proteins comprising the ligand-binding domains of VEGFR1/2 linked to the human IgG1 Fc fragment (Table 1.). These agents suppress angiogenesis by competitively binding VEGF ligands with high affinity, thereby inhibiting VEGFR-mediated signaling pathways. Ziv-aflibercept gained FDA approval in 2012 for metastatic colorectal cancer therapy [266]. Similarly, aflibercept demonstrates significant therapeutic potential in oncology through its VEGF pathway blockade [267].

### HIF- 1 inhibitors

Multiple agents targeting HIF- 1α or associated pathways have shown anti-angiogenic activity (Table 1.) [268]. EZN- 2208 (an irinotecan metabolite), which reduces HIF- 1 mRNA levels and was tested with cetuximab in metastatic colorectal cancer (NCT00931840); CRLX101 (camptothecin-loaded nanoparticles), evaluated in a phase II trial combined with bevacizumab for metastatic renal cell carcinoma (RCC) (NCT02187302); 2-Methoxyestradiol (2ME2), a multitarget HIF- 1α inhibitor, studied alongside sunitinib in metastatic RCC (NCT00444314); 17-AAG (tanespimycin), which destabilizes HIF- 1α via HSP90 inhibition, trialed as monotherapy or with sorafenib in advanced melanoma and solid tumors (NCT00087386, NCT00121264); Vorinostat, a histone deacetylase inhibitor that degrades HIF- 1α, investigated with pembrolizumab in recurrent head and neck squamous cell carcinoma (NCT02538510); PT2977, a second-generation HIF- 2α inhibitor, undergoing phase II evaluation with cabozantinib in advanced RCC (NCT03634540) [232].

### mTOR Inhibitors

Everolimus and Temsirolimus (Table 1.), the mTOR inhibitors, play an important role in the treatment of metastatic RCC. Everolimus, an oral agent, inhibits tumor proliferation and angiogenesis by inhibiting the mTORC1 complex and blocking the PI3 K/AKT pathway. The phase III RECORD- 1 trial showed that its use as a second-line treatment resulted in median PFS (4.0 months vs. Placebo 1.9 months), but ORR was only 1.5%, and median OS was 14.8 vs. 14.4 months [269]. Temsirolimus, an intravenous agent, was evaluated in the phase III Global ARCC trial as first-line therapy for poor-prognosis metastatic RCC (including clear and non-clear cell subtypes). It achieved a median OS of 10.9 months compared to 7.3 months with interferon-α, alongside improved PFS (3.8 vs. 1.9 months). These outcomes led to its recommendation by the NCCN for high-risk patients [270].

### Immunomodulatory agents

Thalidomide and its derivative lenalidomide exhibit significant therapeutic utility in hematologic malignancies (Table 1.). Initially withdrawn due to teratogenicity, thalidomide regained attention for its anti-angiogenic and immunomodulatory properties. It received FDA approval in 1998 for erythema nodosum leprosum and later in 2006 for multiple myeloma based on a phase III trial combining it with dexamethasone [236, 271]. Lenalidomide, an optimized derivative of thalidomide, significantly reduced cytotoxicity by inhibiting tumor cell proliferation and activating IL- 2 release by T cells [237]. In 2013, it was approved for relapsed/refractory mantle cell lymphoma, supported by pivotal phase II trials confirming durable efficacy and safety in treated patients [238].

### Tyrosine Kinase Inhibitors (TKIs)

Sorafenib (Table 1.), an oral multi-targeted TKI, suppresses tumor growth and angiogenesis by targeting VEGFR1/2/3, PDGFR-β, c-Kit, FLT- 3, and inhibiting the Ras/Raf/MEK/ERK signaling cascade [239]. In the phase III SHARP trial, first-line sorafenib significantly extended median OS to 10.8 months compared to 7.9 months with placebo (HR = 0.69) in advanced HCC, securing its approval as the first systemic therapy for this indication [272]. As the first anti-angiogenic TKI, sorafenib has promoted the development of subsequent drugs (such as lenvatinib and regorafenib), and improved the targeting selectivity and safety through structural optimization, which provides an important basis for the evolution of treatment strategies for hepatocellular carcinoma [273].

Sunitinib and pazopanib are multi-targeted TKIs that impede angiogenesis via inhibition of VEGFR- 1/2/3, PDGFR-α/β, and c-Kit (Table 1.). Sunitinib, approved for first-line advanced RCC based on phase III data [241, 274], demonstrated a 1.2-year improvement in median disease-free survival (DFS) post-nephrectomy in high-risk RCC patients in the pivotal NCT00375674 trial, leading to FDA approval in 2017 [275]. Pazopanib, evaluated in the Monk 2010 trial for persistent/recurrent cervical cancer, showed no OS benefit versus lapatinib (HR = 0.96, 95% CI: 0.67–1.38) but modestly prolonged median PFS (18.1 vs. 17.1 weeks) [276]. In addition, the incidence of severe hypertension was significantly higher in the pazopanib-alone group (42.8%) than in the control group (8.3%). Therefore, although pazopanib has shown efficacy in specific tumors (e.g., adjuvant therapy for renal-cell carcinoma), its clinical use needs to be balanced against the risk of adverse effects, particularly monitoring for cardiovascular events [274]. Axitinib, a selective VEGFR- 1/2/3 inhibitor, is primarily utilized in second-line settings, achieving a median PFS of 6.2 months in trials [277]. Despite the emergence of combination immunotherapies (e.g., immune checkpoint inhibitors (ICI) + TKI) as a new standard, sunitinib is still used as first-line therapy in immunotherapy-ineligible patients (e.g., comorbidities or monotherapy), whereas axitinib is used as a second-line option when not involved in first-line regimens [278].

Vorolanib (Table 1.), a highly selective TKI, primarily targets VEGFR and PDGFR. In the randomized, double-blind phase III CONCEPT trial for metastatic RCC, combining vorolanib with the mTOR inhibitor everolimus as second-line therapy significantly extended PFS (10.0 vs. 6.4 months) and improved objective response rates (ORR) (24.8% vs. 8.3%), whereas vorolanib monotherapy demonstrated comparable efficacy to everolimus alone [255]. Although safety profiles were generally tolerable, grade ≥ 3 adverse events occurred more frequently with the combination (72.2% vs. 53.4%). These findings position vorolanib-everolimus as a viable second-line option for patients refractory to first-line VEGFR-TKIs, especially in patients who cannot receive combination immunotherapy or in certain risk strata [279].

Other multi-targeted kinase inhibitors, such as vandetanib, regorafenib, cabozantinib, lenvatinib, Ponatinib, Apatinib, Nintedanib and other small molecule anti-angiogenic drugs (Table 1.), it inhibits tumor proliferation, invasion, metastasis, and angiogenesis by blocking a variety of tyrosine kinase receptors, including VEGFR [10]. Vandetanib, withdrawn for NSCLC due to phase III trial failure but approved in 2011 for advanced medullary thyroid cancer, demonstrating prolonged median OS and PFS [245]. Regorafenib, structurally modified from sorafenib, was approved for metastatic colorectal cancer in 2012 and extended to the second-line treatment of HCC in 2017. The anti-tumor immune regulatory mechanism (p38/Creb1/Klf4 pathway) of regorafenib is an emerging research field [280]. Cabozantinib has been approved for multiple types of angiogensis-related solid tumors due to its potent inhibition of VEGFR- 2/c-Met. Cabozantinib combined with ICI can significantly improve the survival rate of advanced RCC [281]. Lenvatinib, First-line therapy for advanced RCC when combined with everolimus or pembrolizumab (PD- 1 antibody), with ongoing trials exploring its utility in HCC [256]. Although Ponatinib has a broad inhibition effect on angiogenesis kinase, it is only used in leukemia with T315I mutation (ALL/CML) [282]. Apatinib and Nintedanib exhibit clinical promise in diverse solid tumors due to potent kinase inhibition [283, 284].

**Fig. 5:**
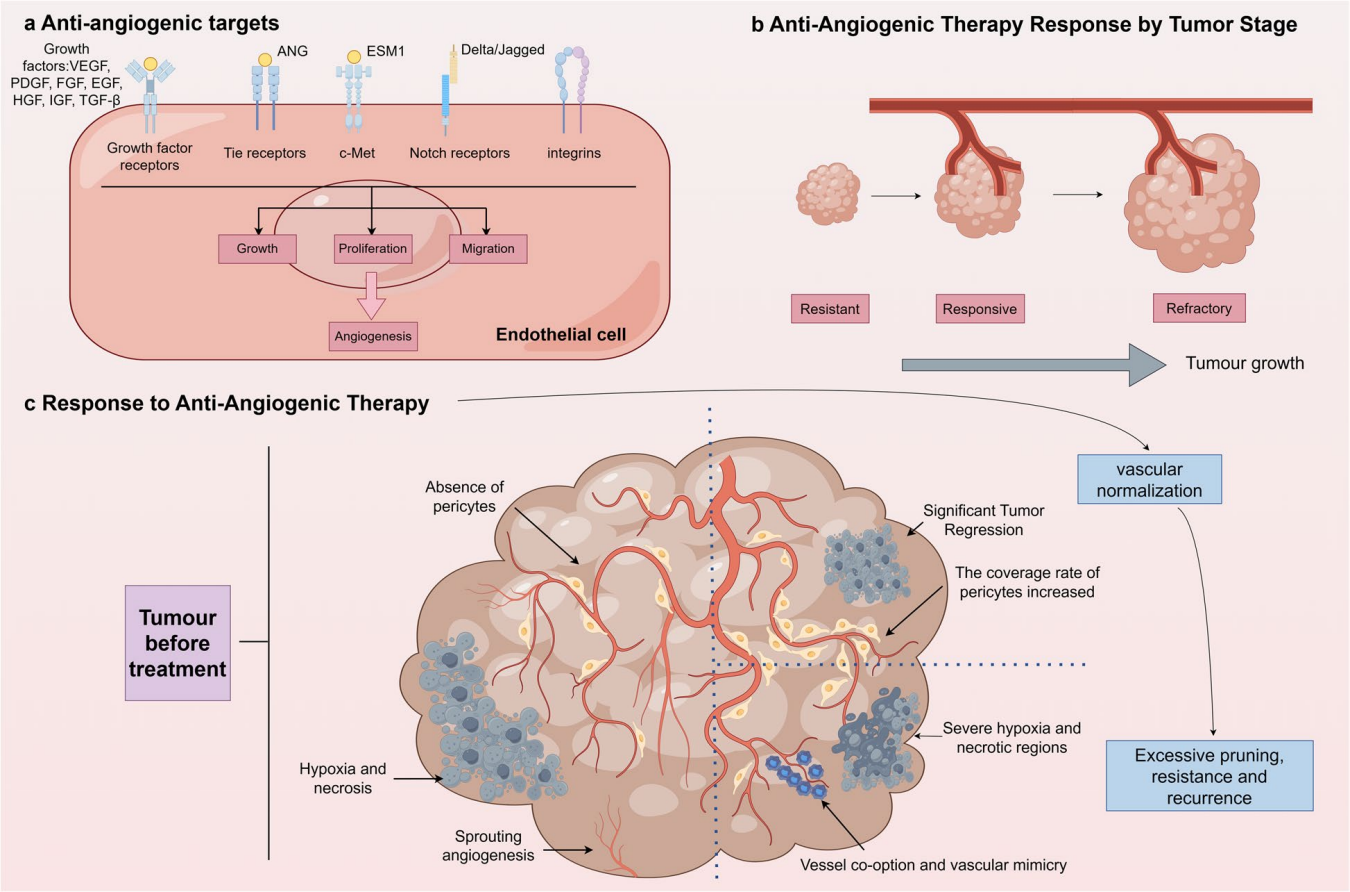
Mechanisms and consequences of anti-angiogenic therapy in tumor vascular targeting. **a** Key molecular targets of anti-angiogenesis include tyrosine kinase signaling pathways (such as VEGF-VEGFR, FGR-FGFR axis, etc.), angiopoietins, ESM1, Notch ligands, and integrins to trigger vessel sprouting by regulating EC proliferation, migration, and survival; **b** The efficacy of anti-angiogenic therapy depends on the tumor growth stage and angiogenesis status, with early tumors being more sensitive to this treatment; **c** The dynamic effect of vascular budding inhibition induces transient vascular pruning that reduces tumor growth through starvation. Moderate anti-angiogenic agents promote vascular normalization (strengthening, stabilizing blood vessels) to improve perfusion and adjunctive therapy. However, excessive pruning can aggravate tumor hypoxia and necrosis, triggering resistance mechanisms such as vascular co-selection, metastasis, and invasive recurrence. Vascular normalization is only a transient phase, as long-term treatment eventually leads to hypoxia-driven tumor progression

Anti-angiogenic inhibitors are employed in cancer therapy to disrupt tumor angiogenesis, but they encounter issues like tumor recurrence, drug resistance, absence of biomarkers, short duration of action, and adverse events [285, 286]. Initially, these inhibitors control tumor growth by blocking blood supply, but prolonged use may increase the risk of invasion and metastasis due to hypoxia. Since drugs targeting angiogenesis primarily interfere with initial vascular development processes such as the multiplication and movement of ECs along with new vessel formation, research findings indicated that newly formed tumors exhibited greater sensitivity to these therapeutic approaches compared to well-developed malignancies at advanced stages (Fig. 5b) [287]. This differential efficacy highlights the importance of early intervention in anti-angiogenic therapy and underscores the challenges in treating established tumors with complex vascular networks [29, 288]. Furthermore, the limited clinical efficacy of anti-angiogenic therapies may stem from alternative vascularization modes such as vascular mimicry and vessel co-option, which bypass traditional angiogenesis pathways and evade therapeutic targeting [289–291]. Concurrently, transient vascular normalization induced by these agents temporarily improves perfusion and enhances adjuvant therapy efficacy, yet ultimately leads to hypoxia-driven relapse (Fig. 5c) [287]. This paradoxical duality underscores the dynamic interplay between vascular remodeling and therapeutic resistance. Drug resistance, whether congenital or acquired, involves complex mechanisms like the upregulation of selective pro-angiogenic pathways and the recruitment of perivascular cells [292–294]. The development of effective biomarkers to monitor treatment response and predict drug resistance is critical, but due to the complexity and variability of tumors, the development of biomarkers faces great challenges. In order to further improve the efficacy of these therapies, more in-depth research and clinical trials are necessary to optimize them.

## Vascular promotion strategies in anti-cancer therapy

Recent advances in vascular regulation have introduced the concept of"vascular promotion"as a counterintuitive but promising approach to improve oncoltherapeutic outcomes. In contrast to conventional anti-angiogenic therapies, which aim to pruritise tumor vasculature, vasopromoting therapies aim to cultivate functional vascular networks to alleviate hypoxia and improve drug delivery [287]. This strategy is based on the premise that transient increases in vessel density and perfusion sensitize tumors to subsequent cytotoxic or immunotherapy interventions [295].

**Fig. 6:**
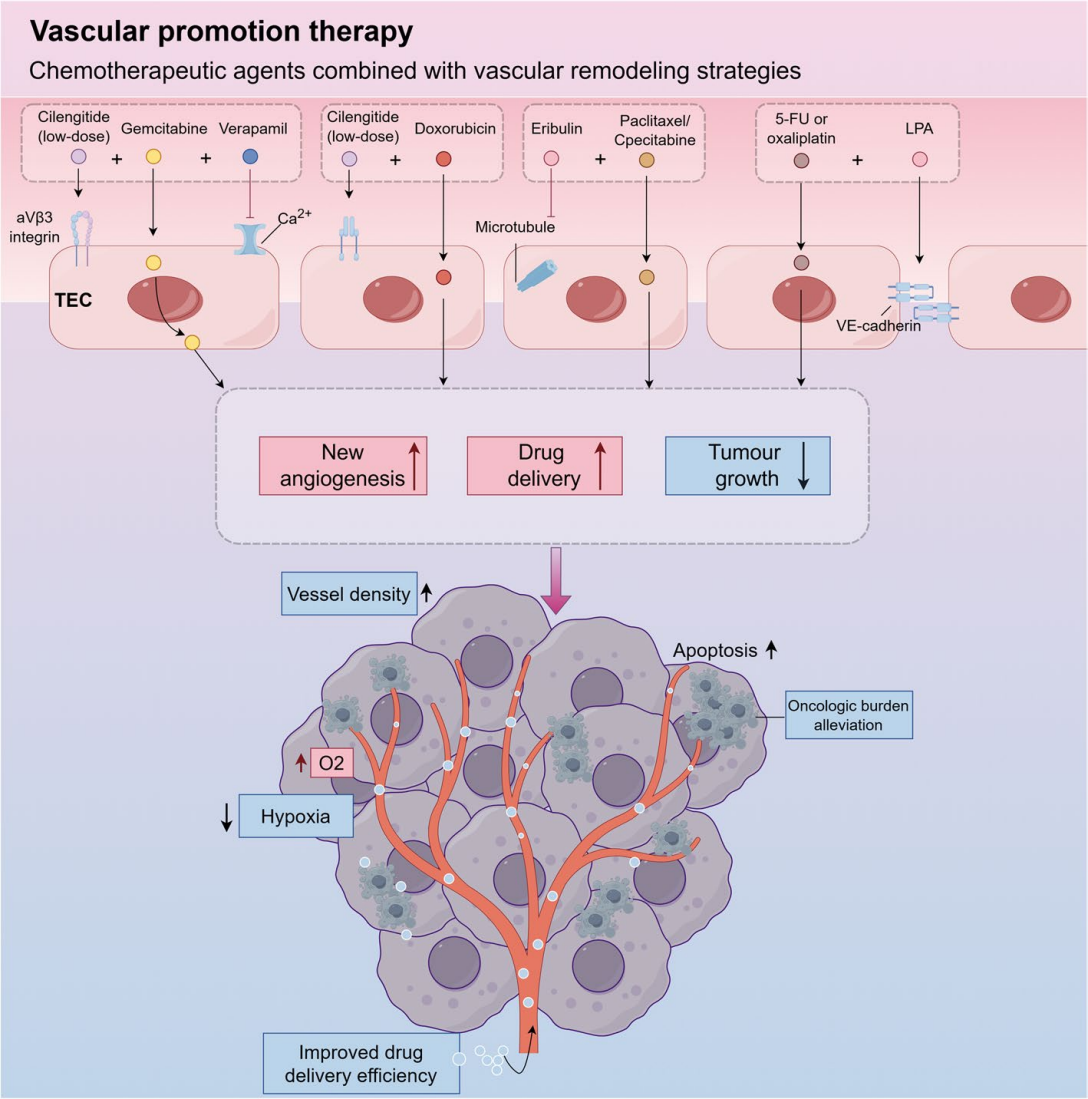
Vascular facilitation Therapy. This strategy promotes functional angiogenesis to improve tumor drug delivery by combining low-dose vascular remodeling drugs with chemotherapy. Low-dose cilengitide, which targets αVβ3 integrin, can increase vascular density and improve perfusion when combined with gemcitabine and verapamil. Epirubicin inhibits microtubule dynamics and, when combined with paclitaxel or capecitabine, reduces hypoxia and enhances antitumor immunity. Lysophosphatidic acid (LPA) activates LPA4 receptor to promote the localization of VE-cadherin, forming a dense vascular network to improve the delivery efficiency of 5-fluorouracil/oxaliplatin

When low-dose integrin inhibitors such as cilengitide are used, low concentrations of cilengitide paradoxically enhance VEGF-mediated signaling by promoting VEGFR2 recycling in ECs, thereby promoting VEGF-mediated angiogenesis [296]. When used in combination with vasodilators such as verapamil and chemotherapeutic agents such as gemcitabine, this approach increases vessel density and blood flow, thereby improving drug penetration into tumor tissue (Fig. 6) [297]. Preclinical studies of this approach in mouse models of pancreatic ductal adenocarcinoma and NSCLC have shown significant inhibition of tumor growth and reduction of metastasis due to enhanced metabolic activation of chemotherapeutic agents within the tumor cells [298]. In addition, an MT1-MMP activated liposome (MC-T-DOX) was designed with low-density cilengitide (αvβ3 integrin targeting peptide). This liposome is digested by MT1-MMP in TECs to release cilengitide, which promotes ECs migration and new angiogenesis, thereby improving tumor blood perfusion and enhancing liposome accumulation in tumors. Subsequently, adriamycin is released by heat trigger to improve drug penetration and cellular uptake and synergistically inhibit tumor growth (Fig. 6) [299]. This strategy significantly inhibited tumor growth with low toxicity in the mouse model of pancreatic cancer, and may be extended to other hypoperfusion tumor treatments in the future.

As vascular promotion strategies were proposed, subsequent studies demonstrated the therapeutic potential of vascular remodeling agents in combination with conventional chemotherapeutic agents. A notable example is aribulin mesylate, a microtubule dynamics inhibitor that exhibits dual antitumor and pro-angiogenic properties in the clinical setting [300, 301]. Experimental evidence from breast cancer and clear cell sarcoma models showed that the combination of capecitabine or paclitaxel not only enhanced vascular density and oxygenation, but also enhanced anti-tumor immune responses, ultimately achieving effective tumor growth inhibition (Fig. 6) [302, 303]. Another innovative strategy involves lysophosphatidic acid (LPA), a bioactive phospholipid that promotes the formation of permeable vascular networks [304]. By activating LPA4 receptor on the surface of TEC, it induces the membrane localization of VE-cadherin at adhesion junctions through Gi and Gα12/13 signaling pathways, thereby enhancing ECs adhesion (Fig. 6). In preclinical models of colorectal and lung cancer, LPA, when combined with 5-fluorouracil or oxaliplatin, increased the permeability of chemotherapeutic agents into tumor tissues by 40% (*p* < 0.001) and significantly inhibited tumor growth (65% reduction in tumor burden in lung cancer model, *p* < 0.001) [305, 306].

In addition, nitric oxide (NO) -based therapies have shown potential to promote angiogenesis. When NO and paclitaxel were co-delivered by TPGS-based polymeric hybrid micellar systems, multiple effects were demonstrated in several mouse tumor models: In situ release of NO can significantly improve tumor vascular permeability, enhance blood perfusion and increase blood vessel density, and then increase drug accumulation through permeability and retention effect and vascular function improvement [307, 308]. At the same time, the synergistic effect of NO and PTX significantly inhibited tumor growth and reduced the risk of metastasis (96% reduction in lung metastatic nodules), and TPGS components further overcame multidrug resistance by inhibiting P-glycoprotein [309].

Despite these advances in vascular promotion therapy, challenges remain in optimizing dosing regimens and identifying biomarkers to monitor therapeutic time Windows [287]. Future work could consider combining vascular promotion with immunotherapy or metabolic inhibitors to amplify the antitumor response. By embedding vascular facilitation in a multimodal framework, this strategy could overcome drug resistance in hypoxic tumors, offering translational potential for patient outcomes.

## Conclusion

The interaction between complex molecular mechanisms and signaling pathways shows rich layers in the process of tumor angiogenesis, which not only reveals its internal fine regulatory network, but also provides many potential targets for the exploration and development of targeted therapeutic methods. Anti-angiogenesis therapy has become one of the common strategies in the field of current anti-tumor therapy. With the deepening of research, scientists continue to explore and find a variety of innovative methods to target the tumor vasculature. Despite the good clinical efficacy of anti-angiogenic therapies, challenges such as drug resistance, adverse reactions, and the need for effective biomarkers remain. Ongoing research is essential for enhancing these therapies, increasing their efficacy, and minimizing side effects, thereby improving patient outcomes and offering hope for more effective cancer treatments. The future of anti-angiogenic therapy lies in continuing to explore the complex tumor microenvironment and developing multi-targeted drugs that combine anti-angiogenic strategies with therapeutic modalities such as immunotherapy to combat drug resistance and enhance treatment response.

## Data Availability

No datasets were generated or analysed during the current study.
